# 

**Published:** 1875-01

**Authors:** 


### CONTENTS:

### ORIGINAL COMMUNICATIONS.

ARTICLE I.—Anæsthesia. By Dr. E. F. Bunnell ........ 385
ARTICLE II.—Extraction of Teeth. By Dr. Eaton ........ 390

### SELECTED ARTICLES.

ARTICLE III.—American Dental Association. (Continued.) ........ 395

ARTICLE IV.—On the Condition of the Mouth and Teeth during Pregnancy. (Continued) By Oakley Coles ........ 410

ARTICLE V.—Salivary Calculus By James A. Salter, F. R. S., Etc, ........ 419

ARTICLE VI.—Physiological Action of Arsenious Acid ........ 423

### EDITORIAL, ETC.

The Southern Dental Association ........ 425
A Warning to Dentists ........ 427
Preparing Vegetable Tissues for the Microscope ........ 427

### MONTHLY SUMMARY.

Intra-Buccal Resection of the Inferior Dental Nerve ........ 428
Treatment of Dog Bites ........ 429
Treatment of Malignant Pustule ........ 430
To Keep Away Flies ........ 431
Influence of Anæsthetics upon the Sexual Impressions of Females ........ 431
Electricity a Substitute for Gas or Coal Oil ........ 431
Ante-natal Development of Nine Teeth ........ 432
Celluloid ........ 432
Handy Method of Examining Nervous Tissues Microscopically ........ 432

